# Monochromatic Green Light Stimulation during Incubation Alters Hepatic Glucose Metabolism That Improves Embryonic Development in Yangzhou Goose Eggs

**DOI:** 10.3390/ijms24010405

**Published:** 2022-12-26

**Authors:** Zhe Chen, Xiaolu Qu, Chungang Feng, Binbin Guo, Huanxi Zhu, Leyan Yan

**Affiliations:** 1 Key Laboratory for Crop and Animal Integrated Farming of Ministry of Agriculture and Rural Affairs, Animal Husbandry Institute, Jiangsu Academy of Agricultural Sciences, Nanjing 210014, China; 2 College of Animal Science and Technology, Huazhong Agricultural University, Wuhan 430070, China; 3 College of Animal Science and Technology, Nanjing Agricultural University, Nanjing 210095, China

**Keywords:** green light, goose egg, hatching, embryo, development, liver, metabolism, muscle, LED

## Abstract

The influence of monochromatic green light stimulation on hatching performance and embryo development has been studied in chickens, but not geese. The liver has crucial functions in the regulation of energy metabolism during embryogenesis, but its involvement in green light transduction is still unidentified. We aimed to determine the influence of monochromatic green light on Yangzhou goose hatching performance and embryo development. We also investigated the metabolomics and transcriptomic responses of the embryonic liver to green light to determine the underlying molecular mechanisms. Eggs were incubated under either 12 h of monochromatic green light/dark (12 L:12D) cycles or 24 h of darkness (0G:24D). Green light promoted embryonic development and hatching performance, also affected the expression of myogenic regulatory factors associated with muscle development. It also shortened hatching time and elevated plasma levels of growth hormone and insulin-like growth factor-1. Metabolomics and transcriptomic results revealed differentially expressed genes and metabolites with enhanced gluconeogenesis/glycolysis and increased plasma glucose and pyruvate levels under green light. Hence, the growth-promoting effect possibly through regulating energy metabolism in the liver and myogenic regulatory factors in muscle. Our findings provide important and novel insights into the mechanisms underlying the beneficial effects of green light on goose embryos.

## 1. Introduction

Commercial fertile eggs are often incubated in the dark in poultry hatcheries. However, eggs are naturally stimulated by light when hens eat and drink. Compared to mammals, birds are more sensitive to environmental illumination because they have four types of single-cone photoreceptors that are responsible for seeing colours, whereas mammals have three [1,2]. Providing light during incubation process is a novel research field, and positively impacts embryonic development [3,4,5,6], especially that from light-emitting diodes (LEDs) in incubators that rarely emit ambient heat [7]. Using LEDs prevents divergence such as accelerated embryonic development induced by overheating under conventional lighting sources. 

Wavelength is one of the three major variables in light management, and green is the most effective color in terms of providing monochromatic light [8,9,10]. Stimulation with green LEDs during incubation shortens the hatching time of broilers and layers by 3.4 and 5.3 h, respectively [9,11], whereas monochromatic green LED stimulation during incubation accelerates embryonic development with an obvious increase in body weight and breast muscle growth [12,13,14,15]. Moreover, monochromatic green light stimulation during incubation induces increased somatotropic axis expression, including hypothalamic growth hormone-releasing hormone (GHRH), plasma growth hormone (GH), hepatic growth hormone receptor (GHR), and insulin-like growth factor 1 (IGF-1) [16,17], especially after 15 days of hatching [10,18]. Therefore, green light has potential applications in commercial incubation processes. However, previous research of photostimulation during incubation has mainly focused on rapidly growing broilers or high-yield laying hens, and only a few have assessed geese. 

Hatchability is considerably lower for fertile goose, than chicken eggs. Low hatchability is attributed in part to poorly developed technology. For example, increasing the turning angle from 45° to 70° or 75° significantly increases hatchability and enhances embryonic growth [19,20]. Optimal lighting programs for geese have been developed to improve egg-laying performance [21,22] and semen quality in ganders [23]. However, suitable lighting programs during goose hatching remains undetermined due to a lack of research. 

The liver plays a crucial role in regulating energy metabolism (lipids and carbohydrates), hormone production, detoxification, bile acid production, protein synthesis, and immunity [24]. The main energy sources for poultry embryos during embryogenesis are lipids that are absorbed from egg yolks, then oxidized in the liver [25]. However, during the late stage of hatching, energy metabolism shifts to glucose metabolism to maintain energy homeostasis for embryonic growth and development [26,27]. The influences of green light stimulation during egg incubation on the development of goslings or the involvement of liver in the transduction process remain unknown. Metabolomics systemically identify and quantify small molecules in biological systems, providing a unique perspective on the changing biological processes [28]. Additionally, liver transcriptome profiling is valuable for characterizing the responses to altered gene expression. Therefore, we aimed to clarify the effects of monochromatic green light during egg incubation on goose embryonic development using multi-omics analyses of hepatic metabolomics and transcriptomic approaches to uncover potential metabolic changes and molecular mechanisms induced by monochromatic light photostimulation during incubation.

## 2. Results

### 2.1. Hatching Time and Hatching Performance

**Figure 1:**
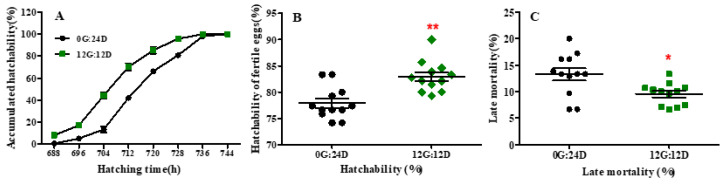
Hatch accumulation and hatch performance of goose eggs under green light and dark. (**A**) Hatch accumulation of eggs. (**B**) Hatchability of fertile eggs. (**C**) Late mortality of goose embryos. Data are presented as mean ± SEM (n = 12 replicates per group at each incubation time). * in red color indicated *p* < 0.05, ** in red color indicated *p* < 0.01. Abbreviations: 0G:24D, dark group; 12G:12D, green light group.

Eggs under monochromatic green light hatched 8 h before those incubated under darkness (733 vs. 741 h; *p* < 0.05) (Figure 1A). The average hatching time was also decreased by green light compared with darkness (710.3 vs. 719.5 h, *p* < 0.01). After 712 h of incubation, approximately 70% of eggs were hatched in the green light group whereas only 42% of eggs were hatched in the dark group. Additionally, the eggs in the green monochromatic light group reached 90% hatching in a shorter period compared to the dark group (*p* < 0.01). However, the hatching window was not significantly affected by green light vs. darkness (45.33 vs. 48 h, *p* = 0. 422). Moreover, hatching performance characteristics were altered in goose eggs exposed to green light during incubation. Green light significantly decreased the mortality rate (*p* < 0.05) of embryos during the late stage of incubation (from embryonic day 19 to 31) (Figure 1B). Consequently, the hatchability of fertile eggs was significantly higher under green light than under darkness (82.95% vs. 77.92%, *p* < 0.01; Figure 1C).

### 2.2. Embryonic Development

**Figure 2:**
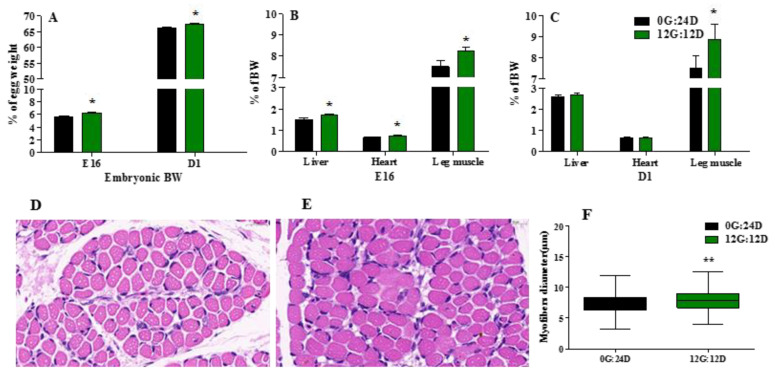
Effect of green light during incubation on embryo and organs development. (**A**) Embryonic BW, as percentage of embryo weight and egg weight incubated under green light or kept in the dark on E16 and D1. (**B**) Liver, heart and leg muscle weight as percentage of BW of on E16. (**C**) Liver, heart and leg muscle weight as percentage of BW of embryos on D1. (**D**,**E**) Image of higher magnifications (magnified 60 times) of representative areas from the gastrocnemius muscle (left) under dark and green light. (**F**) The myofiber diameter of gastrocnemius muscle. The data are presented as mean ± SEM. * *p* < 0.05, ** *p* < 0.01. Abbreviations: dark group, 0G:24D; green light group, 12G:12D; E16, embryonic days 16. D1, day of hatching; BW, body weight.

Green light during incubation significantly enhanced embryonic body weight (BW) calculated as the ratio (%) of embryo to egg weight (Figure 2A) on embryonic day 16 (E16) and the day of hatching (D1) (*p* < 0.05). Exposure to green light in ovo significantly impacted relative heart, liver, and leg muscle weight on E16 (*p* < 0.05), both expressed as a percentage basis of BW (Figure 2B.) Only relative leg muscle weight significantly (*p* < 0.05) differed between the groups on D1 (*p* < 0.05) (Figure 2C).

The diameter of gastrocnemius muscle myofibers was significantly increased on D1 after exposure to green light compared with darkness (Figure 2D,E), in the green light stimulation group, the myofiber diameter was 7.9 μm whereas in the dark group it was only 7.3 μm (*p* < 0.01, Figure 2F).

### 2.3. Plasma GH and IGF-1 Levels

**Figure 3:**
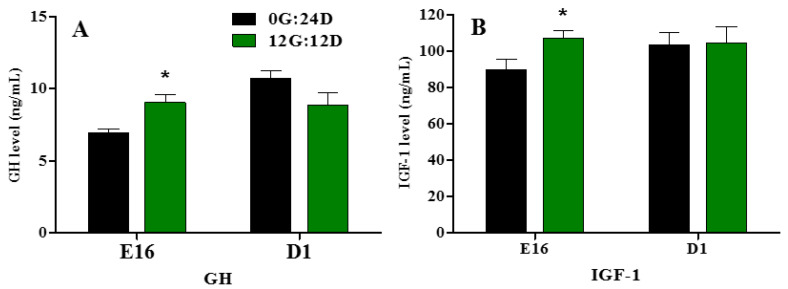
Plasma GH and IGF-1 levels of goose embryos incubated in the dark or under monochromatic green light. (**A**) Plasma GH levels. (**B**) Plasma IGF-1 levels. Data are presented as mean ± SEM (n = 8 embryos per group). Means with asterisks show significant difference between the 2 groups on the same day (* *p* < 0.05).

Embryonic plasma GH and IGF-1 levels significantly increased during embryogenesis at E16 when eggs were incubated under monochromatic green light (*p* < 0.05, Figure 3A,B). However, on D1, no significant differences in plasma GH and IGF-1 levels were observed between the two groups. 

### 2.4. Expression of Genes Associated with Leg Muscle Development

**Figure 4:**
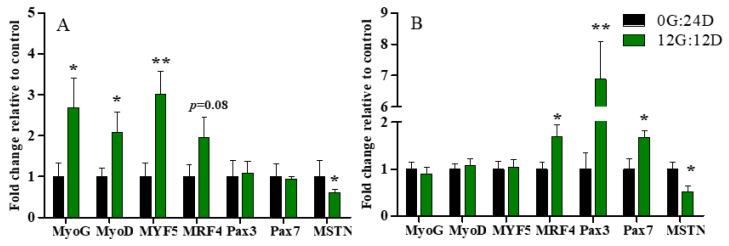
Relative expression of mRNA in leg muscles of goose embryos incubated in the dark or under monochromatic green light. Panels (**A**,**B**) represent relative abundances of mRNA for *MyoG*, *MyoD*, *MYF5*, *MRF4*, *Pax3*, *Pax7* and *MSTN* on E16 and D1, respectively. Data are presented as mean ± SEM (n = 8 embryos per group). * *p* < 0.05, ** *p* < 0.01.

The expression of genes associated with leg muscle development differed between the two groups at various developmental stages (Figure 4). The expression of myogenin G (*MyoG*), myogenic differentiation antigen (*MyoD*)*,* and myogenic factor 5 (*MYF5*) mRNA was, respectively, ~2.68-, 2-, 3-fold enhanced under green light than darkness on E16, (*p* < 0.05, *p* < 0.05, and *p* < 0.01, respectively; Figure 4A), but did not significantly differ on D1 (*p* > 0.05, Figure 4B). Moreover, green light during incubation tended to upregulate *MRF4* mRNA expression, but the difference did not reach statistical significance (*p* = 0.08). Gene expression of *MRF4* was significantly higher in embryos on D1 incubated under green light than darkness (*p* < 0.05). 

Green light did not change paired box 3 (*Pax3*) and *Pax7* mRNA expression on E16 (*p* > 0.05, Figure 4A), but significantly increased it on D1 (*p* < 0.01 and (*p* < 0.05, respectively; Figure 4B). In addition, Myostatin (*MSTN*) RNA expression significantly declined in embryos incubated under green light compared with darkness on E16 and D1 (*p* < 0.05; Figure 4A,B).

### 2.5. Global Transcriptomics Profiles in the Embryonic Livers

**Figure 5:**
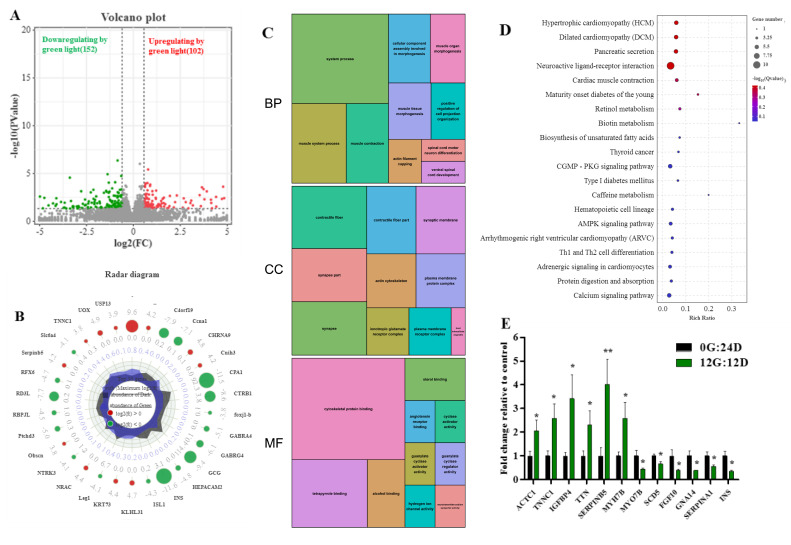
The effect of the green light stimulation during incubation on global transcriptomics profiles in the liver of goose on embryo day 16 (E16). (**A**) Volcano plots indicating significant DEGs in the liver of the green light (G) and the dark (D) treatments. Red and green dots are the up- and downregulated of DEGs caused by green light (G vs. D, n = 5), respectively. (**B**) Radar diagram showing the 30 maximum folding change DEGs in the liver on E16. The green circles are log2-transformed fold change <0, the red circles are log2-transformed fold change >0. (**C**) The significantly enriched categories of gene ontology (GO) terms of the DEGs. (**D**) The top twenty-five significantly enriched KEGG pathways of the DEGs. (**E**) Validation of the gene expression profile by real-time PCR (n = 8). * indicates *p* < 0.05, ** *p* < 0.01.

We investigated the profiles of differentially expressed genes (DEG) among 15,455 genes between liver tissues in goose embryos on day 16 to determine the effects of green light. We identified 254 DEGs between eggs incubated under green light and darkness (FDR adjusted *p* < 0.05; log2 (fold change|FC|) ≥ 1), of which 102 and 152 were, respectively, upregulated and downregulated. Figure 5A shows volcano plots of the DEGs between the two groups indicating distinct transcriptional profiles. Figure 5B shows maximally 30-fold changed DEGs on E16 in the livers of embryos incubated under green light and darkness groups during incubation.

Functional categories were determined using GO annotation (Figure 5C). Among the categories of biological process, the most significantly enriched terms were muscle system process, muscle tissue morphogenesis, and muscle contraction. The most enriched GO terms in molecular functions were associated with cytoskeletal protein binding and tetrapyrrole binding. Moreover, the cellular component of GO for the liver was enriched in contractile fiber, synapse part, and actin cytoskeleton. These results suggested that green light upregulates the expression of genes associated with muscle development.

The Kyoto Encyclopedia of Genes and Genomes (KEGG) results revealed that the DEGs were associated with multiple important pathways. The bubble chart in Figure 5D displays the top 25 KEGG pathways. Those affected by green light during goose egg incubation comprised hypertrophic cardiomyopathy, dilated cardiomyopathy, pancreatic secretion, cardiac muscle contraction, pancreatic secretion, biosynthesis of unsaturated fatty acids, protein digestion and absorption, and cGMP-PKG signaling pathways. These results indicated that green light regulates muscle development.

We analyzed 12 genes to confirm the expression profiles and validate the transcriptome results (Figure 5E) and found that all gene expression profiles were in line with those from transcriptome sequencing. Therefore, indicating our data and the transcriptome sequencing platform were reliable.

### 2.6. Global Metabolomic Profiles in Embryonic Livers

**Figure 6:**
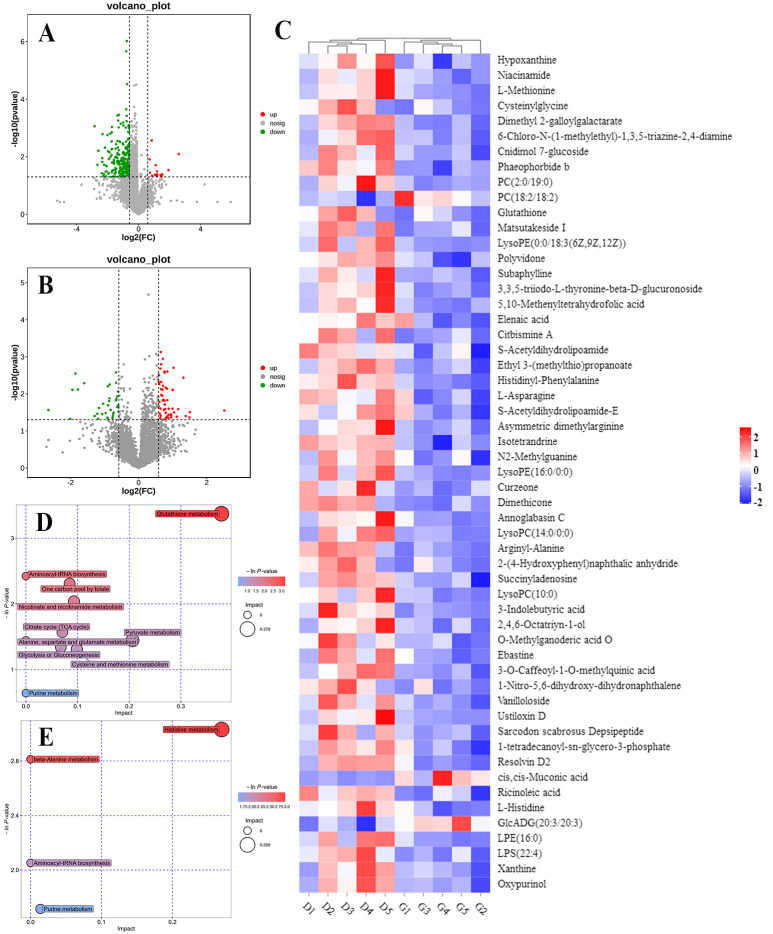
The effect of the green light stimulation during incubation on global metabolomics profiles in the liver. POS, positive ion mode; NEG, negative ion mode. (**A**,**B**) Volcano plot showing the DEMs in the liver of the green light and the dark group from the positive and negative ion modes. (**C**) The heatmap analysis of DEMs between two groups. (**D**,**E**) The significantly changed metabolic pathways from the positive and negative ion models, respectively. The size and color of each circle represent pathway rich-factor and *p*-value, respectively.

We profiled and compared the metabolomes of livers obtained from embryos incubated under green light and darkness to further understand the molecular mechanisms underlying altered gene expression. We identified 276 (15 upregulated and 261 downregulated) and 146 (94 upregulated and 52 downregulated) differentially expressed metabolites (DEMs), based on the criteria of *p* < 0.05 and VIP > 1 in positive and negative modes, respectively, between the two groups. Volcano plots show regulated metabolites between the two groups (*p* < 0.05, FC > 1.5 or < 0.67) in positive and negative ion modes, respectively; Figure 6A,B. Figure 6C shows a metabolite heatmap of 55 DEMs. Most of these were downregulated and only phosphatidyl choline (PC; 18:2/18:2), cis,cis-Muconic acid, and GlcADG (20:3/20:3) were upregulated in liver samples from embryos incubated under green light compared with darkness. 

To understand the pathways influenced by green light during incubation, we annotated DEMs from the two groups according to the KEGG pathways. The results revealed that green light significantly altered several metabolic pathways in embryonic livers, in both positive and negative modes (*p* < 0.05; Figure 6D,E). S-acetyldihydrolipoamide-E, a common intermediate product of glycolysis or gluconeogenesis, citrate cycle (TCA cycle), and pyruvate metabolism pathways were significantly downregulated in the embryos incubated under green light compared with darkness. Additionally, L-asparagine, L-methionine, and L-histidine involved in aspartate/methionine/histidine metabolism and aminoacyl-tRNA biosynthesis pathways were also downregulated.

### 2.7. Integrated Analyses of the Metabolomics and Transcriptomics Data

**Figure 7:**
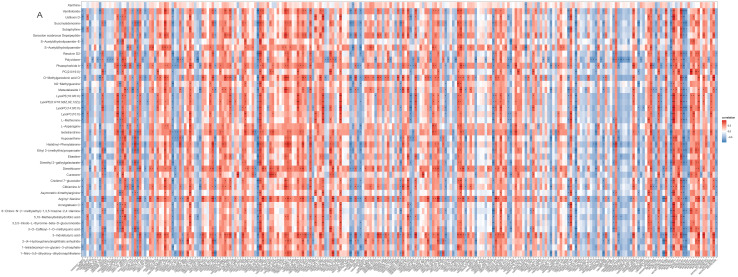

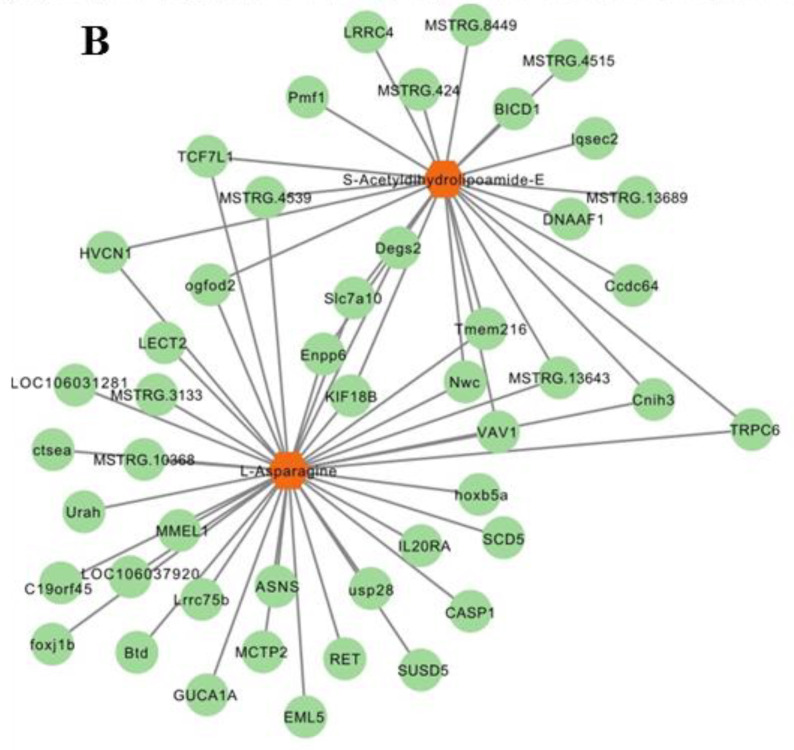
Integrated enrichment analysis of metabolites and DEGs. (**A**) Heatmap of all DEMs and DEGs. Gene names were described in italics and metabolites names in normal font. * indicates *p* < 0.05, ** *p* < 0.01, *** *p* < 0.001. (**B**) The DEGs related to s-acetyldihydrolipoamide-E and L-asparagine. Metabolites are in orange hexagon. Each green circle represents a gene.

A heatmap shows that the expression of most DEMs and DEGs closely correlated (*p* < 0.05; Figure 7A). S-acetyldihydrolipoamide-E and L-asparagine that are involved in pyruvate metabolism and gluconeogenesis/glycolysis correlated with all relevant DEGs (Figure 7B) including asparagine synthetase (*ASNS*), stearoyl-CoA desaturase 5 (*SCD5*), ectonucleotide pyrophosphatase/phosphodiesterase 6 (*ENPP6*), and proto-oncogene vav-like (*VAV1*).

### 2.8. Biochemical Index Related to Glucose Metabolism

**Figure 8:**
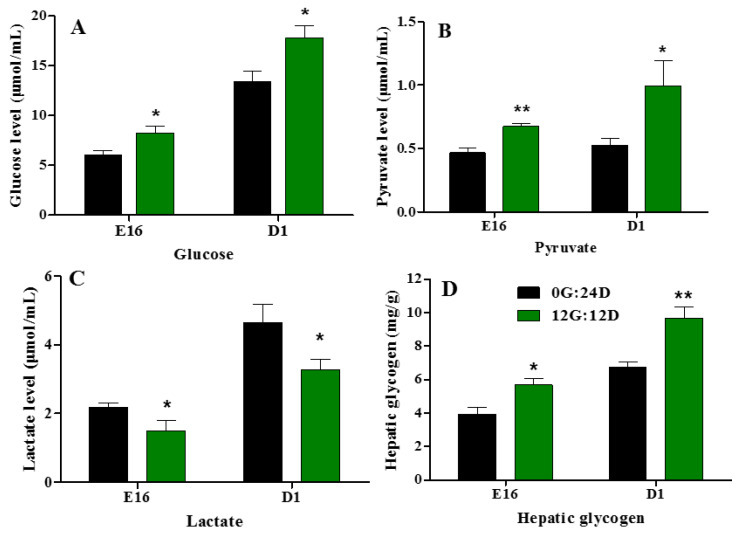
Plasma glucose, pyruvate and lactate levels of goose embryos incubated in the dark or under monochromatic green light. (**A**) Plasma glucose levels. (**B**) Plasma pyruvate levels. (**C**) Plasma lactate levels. (**D**) Hepatic glycogen levels. Data are presented as mean ± SEM (n = 8 embryos per group). Means with asterisks show significant difference between the 2 groups on the same day (* *p* < 0.05, ** *p* < 0.01).

Plasma glucose, pyruvate, and lactate levels increased (Figure 8A–C) during goose embryogenesis. Monochromatic green light photostimulation during incubation significantly upregulated glucose, pyruvate, and lactate levels in embryonic plasma on E16 and D1 (*p* < 0.05, Figure 8A,B). However, plasma lactate levels were significantly lower in the group incubated under green light than darkness on E16 and D1 (*p* < 0.05, Figure 8C). Hepatic glycogen increased as the incubation lengthened in the group incubated under green light, compared with the darkness on E16 and D1 (*p* < 0.05 and *p* < 0.01, respectively; Figure 8D).

## 3. Discussion

Different sources and spectra of light are regarded as important environmental factors in poultry production. It is widely applied in the commercial production of geese, but not during the incubation period. This is a crucial phase in the life of poultry, and manipulating incubation conditions can benefit hatchability, chick quality, embryonic development and growth [29,30]. Here, we investigated the effects of green light during incubation on the timing and performance of hatching, and on the development of Yangzhou goose embryos. Green light significantly promoted gosling growth, increased liver and leg muscle weights and elevated plasma GH and IGF-1 levels. Moreover, hepatic metabolomic and transcriptomic analyses indicated that green light induces changes in glucose metabolism, manifested as enhanced gluconeogenesis during embryogenesis.

Total and average hatch time, and time reaching 90% hatch are important indicators of hatching characteristics. Here, green light during incubation shortened all of these parameters, which was consistent with previous findings showing that LED light during embryogenesis decreased the average hatching time of broilers by 3.4 h [9]. Generally, reduced hatching time can lead to energy conservation for post-hatch growth [11]. Nevertheless, chicks that hatch early during a wider window, might have delayed access to food and water [31,32]. A scattered hatching period can also impair the growth of hatched chicks and post-hatched embryos [33]. We found that green light in ovo did not influence the hatching window. Others have similarly found that green light reduces the hatching time for layers but does not widen the hatch window [11,34].

The present study found significantly lower late mortality rates and significantly increased hatchability of fertile goose eggs in the group incubated under green light than darkness. However, green LED light did not influence hatchability in turkeys [4], layers [11], or broilers [14,35], although a study discovered a significant decrease in hatchability during green light incubation [9]. The discrepancies among these findings might have been due to differences in light intensity, photoperiod, and absence of light–dark cycle, or the light absorption capacity of eggshells, such as color, strength and thickness. Collectively, our results indicate that exposure to green LED light during incubation advanced the time of hatching by 6 h, but did not diminish the hatching performance of goslings.

Green LED light during embryogenesis in ovo positively impacts embryonic development, manifested by increased pectoral muscle and embryo weight [12,15,32]. A shorter hatching period is associated with increased hatching weight and post-hatching growth [36]. We showed that monochromatic green light significantly increased relative leg muscle growth and body weight, as well as muscle fiber diameter and size from E16 until D1, which was consistent with these findings. Fiber diameter inversely correlates with the total number of muscle fibers which substantially determines the capacity of postnatal muscle growth. Therefore, the reduced diameter of leg muscle fibers on D1 in the green light group suggests muscle developmental potential during the early post-hatch stage. Notably, intermittent lighting (30 min on and 30 min off) avoids overheating the eggs and eliminates the effects of heat on embryo development.

The somatotropic/pro-growth axis controls muscle growth and development, which includes hypothalamic GHRH, GH from the anterior pituitary gland, and IGF-1 derived from the liver and skeletal muscles. Both GH and IGF-1 participate in the proliferation and differentiation of myoblasts [37] and satellite cells [38,39]. In addition, GH functions can be direct or indirect through IGF-1 synthesis [40]. Several embryonic tissues express IGF-1 mRNA during the early incubation stage, and IGF-1 plays an important role in accelerating embryonic development [41]. Increased IGF-1 and GH levels are associated with embryonic and post-hatch growth in broilers [42]. Green light during incubation positively affects GHRH, GH, and IGF-1 levels in broilers [16,17,18,38]. Consistent with these facts, the present study identified increased GH and IGF-1 production during embryonic development, confirming that green light promotes embryonic development, accompanied by increased plasma GH and IGF-1 levels.

Skeletal muscle development (myogenesis) is tightly controlled by muscle-specific myogenic regulatory factors such as MyoD, MYF5, MyoG, and MRF4. The expression of these factors is undetectable in quiescent satellite cells, but they are sequentially expressed when these cells are activated [30,43]. Satellite cells (myoblasts) under Pax3 and Pax7 regulation begin the myogenic program by expressing MYF5 and MyoD [44]. When satellite cells are activated to generate daughter myogenic precursor cells, MyoG and MRF4 subsequently regulate terminal differentiation into myotubes [45]. In contrast, MSTN negatively regulates skeletal muscle proliferation and differentiation and downregulates Pax 3, MyoD, MYF5 and MyoG expression [46,47]. The expression of MRF genes including MyoG and MRF4 is upregulated by IGF-1 administered to duck embryos in ovo [48], whereas Pax7, MYF5, and MyoD is upregulated, and MSTN expression is downregulated in embryonic chickens [37]. We found significantly higher *MyoG, MyoD*, and *MYF5* mRNA expression in leg muscles accompanied by upregulated IGF-1 production in embryos incubated under green light than darkness on E16 but not on D1. Moreover, green light stimuli upregulated *MRF4* mRNA expression on D1. Green light during incubation also upregulates *MyoG* and *MyoD* mRNA expression, resulting in increased muscle fiber and chicken breast muscle development one week after hatching [43]. This phenomenon indicates that green light promotes the proliferation and differentiation of skeletal muscle satellite cells during late embryogenesis, and that satellite cells start to proliferate before differentiation. In contrast, decreased *MSTN* expression under green light, confirmed its role in counteracting muscle growth. In addition, *Pax3* and *Pax7* mRNA expression was significantly increased under green light on D1. Paired box 7 is considered to be an early marker of myogenesis during post-hatching muscle growth [49]. Therefore, these results suggest that monochromatic green light photostimulation results in a larger reservoir of myogenic progeny cells in newly hatched goslings, and further explains the stimulated growth of embryonic leg muscle and the larger myofiber diameter on D1. 

Insulin-like growth factor 1 is mainly secreted in the liver, where GHRs are located in the liver, which is the connection between the somatotropic axis and green light photostimulation. Liver development was accelerated under green light. Energy metabolic pathways, such as gluconeogenesis, glycolysis, and glycogenesis, are upregulated in the liver during the final critical period of embryo development [50]. Our transcriptome results showed that the expression of genes involved in muscle development, including muscle system process, muscle tissue morphogenesis, and muscle contraction, was induced by green light during incubation. In addition, green light influenced several metabolic pathways, including amino acid metabolism, the citrate cycle (TCA cycle), pyruvate metabolism, glycolysis, and gluconeogenesis. Asparagine synthetase (ASNS) is a key enzyme that catalyzes the synthesis of asparagine from aspartate [51]. Both *ASNS* transcript and L-asparagine levels were downregulated by green light, resulting in an increased aspartate reserve in the liver, which might provide more substrate for pyruvate synthesis. As an intermediate product during the synthesis of acetyl-CoA from pyruvate [52], less s-acetyldihydrolipoamide-E was found in the liver under green light than darkness, indicating increased pyruvate production in the liver. Furthermore, the biochemical indices also confirmed these results, as pyruvate levels were increased by green light. The increase in hepatic glycogen and parallel change in serum glucose content suggest that green light enhances glucose synthesis/gluconeogenesis in the liver, thereby increasing glucose content.

Energy metabolism from yolk lipids switches to anaerobic glucose catabolism and gluconeogenesis during the late hatching stage when the oxygen consumption of embryo in ovo reaches a specific threshold [50,53]. Pyruvate is converted into lactate during anaerobic glycolysis [54]. Here, the lactate content increased from E16 to D1, indicating enhanced glycolysis during embryo development. However, an increase in plasma pyruvate with a decline in lactate content indicated that glycolysis was inhibited under green light compared with darkness. Gluconeogenesis is the formation of new glucose from common physiological metabolites. Upregulated gluconeogenesis is crucial in the later-term embryo to ensure a supply of blood glucose for growth and development, as well as muscle and liver glycogen deposition [26]. Blood glucose levels positively correlate with embryonic survival rates and hatchling weight [55]. The present study found that green light during incubation in ovo altered liver glucose metabolism, which was manifested by enhanced glucose synthesis and suppressed glycolysis. This led to increased energy production to satisfy the high-energy demand during the late stage of embryogenesis. Increased plasma glucose levels in embryos also trigger glycogen synthesis in muscle, which is conducive to muscle development. Therefore, embryo development and mortality were, respectively, accelerated and decreased when embryos were incubated under green light compared with darkness. Moreover, IGF-1 plays a crucial role in carbohydrate, lipid, and protein metabolism in liver and muscle [56] and stimulates hepatic glycogen synthesis [41]. Therefore, IGF-1 secreted by the liver exerts positive feedback regulatory effects on hepatic glucose metabolism. Changes in environmental factors (temperature, humidity and light exposure) and nutritional resources after hatching are significant challenges for young goslings. Our study revealed that green light increases glucose levels, which result in more stored energy for the survival of newly hatched goslings. Therefore, green light during incubation might facilitate the adaptation of goslings to the post-hatch environment.

## 4. Material and Methods

### 4.1. Eggs and Experiments

The Research Committee of Jiangsu Academy of Agricultural Sciences approved the study and all experimental procedures complied with the Regulations for the Administration of Affairs Concerning Experimental Animals (Decree No. 63 of the Jiangsu Academy of Agricultural Science; 8 July 2014).

The same number of Yangzhou goose eggs were assessed in three consecutive incubations. We collected 1536 normal eggs (average weight, 143.33 ± 5.43 g) from 40-week-old Yangzhou geese (male: female = 1:5) and analyzed 512 eggs per experiment. The eggs were stored no more than 3 d with a temperature of 20°C. The front windows of two incubators (FHQ-V1, Hengxin Incubation Equipment Co., Ltd., Nanjing, China) were covered with opaque sheets to prevent light intrusion from outside. One incubator was fitted with strips of monochromatic green LEDs (NVC Lighting Co., Ltd., Guangzhou, China), with a 12 h green light/12 h dark cycle (12G:12D) during the first 28 days of incubation. We eliminated light-induced egg overheating using a mechanical timer to control 24 green-light cycles of 0.5 h of darkness followed by 0.5 h of light. Light intensity at the egg level was adjusted to 200 lx using a digital luxmeter. Eggs in the other incubator remained in complete darkness (0G:24D). Thereafter, 512 eggs were equally assigned to the same location in eight trays per incubator and every two trays were replicates.

The eggs were initially incubated at 38 °C, then this was reduced by 0.2 °C–0.3 °C every 5–10 d. The relative humidity at each stage was 63%, 58%, 55%, 65% and 70% and the eggs were turned 45° once every 2 h. The eggs were candled on days 7 and 18, and cooled with water at 38 °C for 5 min per day from incubation days 9–28. Unfertilized eggs and those containing dead embryos were removed, then the viable eggs were transferred to the hatcher on day 28 without further turning. 

Hatched goslings were counted every 8 h from 688–744 h after incubation, then hatch time, time reaching 90% hatch, and average hatch time were quantified for all goslings. Average hatch time was defined as the total hatching time of all goslings divided by the total number of goslings. Time to 90% hatch was the amount of time required for 90% of the goslings to hatch. The hatch window was defined as the interval from the emergence of the first to the last gosling. In addition, the early (from embryonic day 1 to 7), intermediate (from embryonic day 8 to 18) and late mortality (from embryonic 19 to 31) of goose embryos were also calculated.

### 4.2. Tissue Preparation

We randomly selected 10–13 fertilized eggs from both groups at the end of embryonic day 16 (E16) and the day (D1) when the third batch of incubated embryos hatched. The eggs were weighed, and opened, the embryos were cleaned of external membranes and weighed. The livers, hearts and left leg muscles were isolated and weighed to track organ development. Five liver samples at E16 were rinsed with cold saline, immediately frozen in liquid nitrogen and stored in −80 °C. 

### 4.3. Histological Assessment

Leg muscle tissues were histologically assessed as described [19]. At least six arbitrary fields in two serial sections of each muscle sample were photographed using a ×60 objective. The diameters of ~1000 individual myofibers was then determined in eight muscle sample using Image J v1.8.0 (National Institutes of Health, Bethesda, MD, USA). 

### 4.4. Transcriptomic Analysis

Total RNA of liver tissue (approximately 15–30 mg) was extracted using TRIzol^®^ reagent kits (Invitrogen, Carlsbad, CA, USA) as described by the manufacturer. After quality assessment and enrichment using Oligo (dT) beads, the mRNA was reverse transcribed into cDNA. Double-stranded cDNA was subsequently constructed using a cDNA library and an Illumina NovaSeq 6000 at Gene Denovo Biotechnology Co., Ltd. (Guangzhou, China). Clean reads were mapped to the reference *Anser cygnoides* genome by HISAT2. 2.4 and assembled using StringTie v1.3. 

Gene abundance was quantified as fragments per kilobase of transcript per million mapped reads (FPKM) using RSEM software. We compared DEGs between the two groups using DESeq2 software. Genes with the parameter of false discovery rate (FDR) below 0.05 and absolute fold change ≥1.5 were considered differentially expressed genes (DEGs). 

We analyzed Gene Ontology (GO) enrichment using an online platform (http://www.geneontology.org/, accessed on 22 October 2021), as well as all annotated genes in the *Anser cygnoides* genome as background and KEGG pathways (http://www.genome.jp/kegg/, accessed on 25 October 2021). Gene Ontology terms and pathways were defined as significantly enriched by DEGs if they met FDR ≤ 0.05.

### 4.5. Metabolomics Analysis

Untargeted metabolomics were analyzed at Biotree Ltd. (Shanghai, China). Briefly, five randomly selected liver samples (~25 mg) from the two groups were incubated in methanol: water (3:1) containing an isotopically-labelled internal standard, then homogenized, sonicated in an ice-water bath. After being set at −20 °C for 1 h, the samples were centrifuged at 12,000 rpm, 4 °C for 15 min, and supernatants were analyzed by liquid chromatography–tandem mass spectrometry (LC-MS/MS) using a Vanquish UHPLC system with an Orbitrap MS UPLC HSS T3 column (both from Thermo Fisher Scientific Inc., Waltham, MA, USA). We acquired MS/MS spectra using a QE HFX mass spectrometer in information-dependent acquisition mode controlled by Xcalibur acquisition software (Thermo Fisher Scientific Inc.). The original data were converted to the mzXML format using ProteoWizard, then processed for peak detection, extraction, alignment, and integration using the R package XCMS. Thereafter, metabolites were annotated using an in-house MS2 database.

After preprocessing the original data, OPLS-DA (orthogonal projections to latent structures-discriminate analysis) was applied to process the metabolomic data using SIMCA V15.0.2 (Sartorius Stedim Data Analytics AB, Umea, Sweden). Significantly differential metabolites were identified when the variable importance for the projection (VIP) was > 1 and *p* < 0.05 in Student *t*-tests. Pathway enrichment was assessed using KEGG (www.genome.jp/kegg/, accessed on 12 April 2022) and MetaboAnalyst (http://www.metaboanalyst.ca/, accessed on 13 April 2022).

### 4.6. Quantitative Real-Time PCR (qRT-PCR)

We validated DEGs identified by transcriptome sequencing using quantitative reverse transcription-polymerase chain reaction (qRT-PCR). We randomly selected the following DEGs: actin alpha cardiac muscle 1 (ACTC1), troponin C1 (TNNC1), insulin-like growth factor binding protein 4 (IGFBP4), titin (TTN), serpin family B member 5 (SERPINB5), myosin heavy chain 7B (MYH7B), myosin VIIB (MYO7B), SCD5, fibroblast growth factor 10 (FGF10), guanine nucleotide binding protein alpha 14 (GNA14), serpin family A member 1 (SERPINA1), and insulin (INS). Table 1 shows the primer sequences for these genes. Total RNA (1 μg) isolated from liver tissues (n = 8) using RNAprep Pure Cell Kits (Tiangen Biotech, Beijing, China) was transcribed into cDNA using PrimeScript™ RT Reagent Kits (Perfect Real Time) (Takara Bio Inc., Kusatsu, Japan) as described by the manufacturer. Quantitative RT-PCR proceeded in 20-μL volumes using a SYBR^®^ Premix EX Taq Kit (Takara Bio Inc.) as described by the manufacturer under these conditions: 2 min at 95 °C, followed by 40 cycles of 10 s at 95 °C, 30 s at 60 °C, and 30 s at 72 °C. Amplicons were detected using ABI 7500 system (Applied Biosystems, Foster City, CA, USA). Threshold cycle (Ct) values were calculated using ABI 7500 software V.2.0.6. Relative expression of the selected genes was calculated using the 2^−ΔΔCT^ method and normalized to the stably expressed housekeeping gene (*β-Actin*) [57]. The stability of *β-Actin* between the dark and green light group was confirmed. The relative abundance of *MyoD*, *MYF5*, *MyoG*, myogenic regulatory factor-4 (*MRF4*), *Pax3*, *Pax7*, and myostatin (*MSTN*) mRNA that regulate myogenesis in leg muscle were also quantified using glyceraldehyde-3-phosphate dehydrogenase (*GAPDH*) as an endogenous control. 

### 4.7. Biochemical Analysis

Blood samples (1–2 mL) collected from the chorioallantoic vessel of goose embryos into a heparinized syringe on E16 and D1 were centrifuged at 1000× *g* at 4 °C for 15 min. Plasma was aspirated and stored in conical vials at −80 °C.

Plasma GH concentrations were measured using an in-house goose GH ELISA that we recently developed [58]. Assay sensitivity was <0.1 ng/mL, and the intra- and inter-assay coefficients of variation (CVs) were 10% and 13%, respectively. Plasma IGF-I concentrations were assayed using ELISA kits (MLBio, Shanghai, China). The intra- and inter-assay CVs were <10% and 15%, respectively. The contents of glucose, pyruvate and lactate in plasma and glycogen liver were measured using appropriate kits Nanjing Jiancheng Bioengineering Institute, Nanjing, China) as described by the manufacturer.

### 4.8. Statistical Analysis

Data, including the hatchability of fertile eggs, embryonic mortality, E16 body weight (BW), hatching body weight, relative liver weight (liver weight/body weight), relative heart weight (heart weight/body weight), and relative leg muscle weight (leg muscle weight/body weight) were analyzed by Student’s *t*-tests using SPSS v. 18.0 (IBM Corp., Armonk, NY, USA). The RT-qPCR results are expressed as the means ± SEM of eight samples per group analyzed using Student’s *t*-tests. Differences were considered significant at *p* < 0.05.

## 5. Conclusions

**Figure 9:**
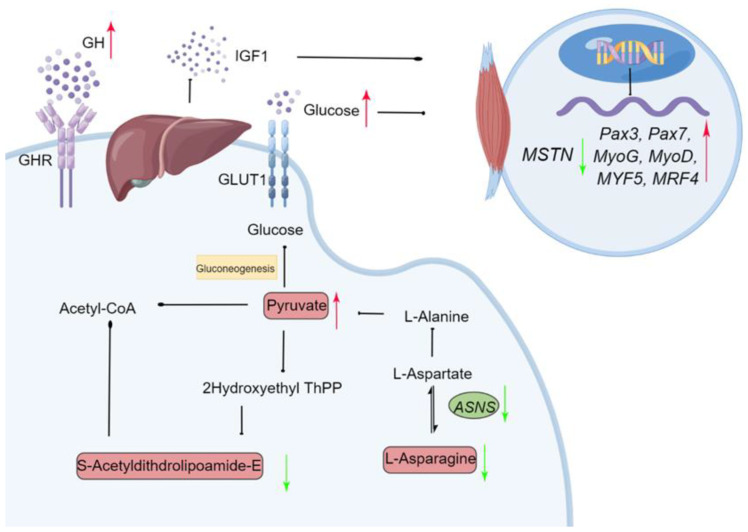
Schematic diagram of the proposed molecular mechanism for monochromatic green light photostimulation during incubation induced improving embryonic development in Yangzhou goose eggs. Green arrow (downregulated) and red arrow (upregulated) in the green light vs. the dark treatments. Gene names were described in italics and metabolites names in normal font. ASNS: Asparagine synthetase; GLUT1: Glucose transporter 1; GHR: Growth factor receptor.

Monochromatic green light promoted IGF-1 secretion from the livers of geese via GH, then enhanced hepatic gluconeogenesis to satisfy a high energy demand during late embryogenesis. Glucose and IGF-1 also affected myogenic activity by regulating the expression of MRFs to promote muscle and embryonic development. Moreover, monochromatic green light improved goose embryonic development and hatching performance. These results suggested that the embryonic development and growth-stimulating effects of green light during incubation are associated with the regulation of energy metabolism and the interactions with MRFs related to growth control. This study provides important novel insights into understanding the mechanisms that are triggered by monochromatic green light and enhance embryo development (Figure 9).

## Figures and Tables

**Table 1:** The primer sequences for q-PCR.

Genes	Accession Number	Primer Sequence (5′–3′)	Fragment Size (bp)
*β-Actin*	XM_048058703.1	TGACGCAGATCATGTTTGAGA GCAGAGCGTAGCCCTCATAG	159
*GAPDH*	XM_013199522.2	GCTGATGCTCCCATGTTCGTGAT GTGGTGCAAGAGGCATTGCTGAC	86
*TNNC1*	XM_013191946.2	GGAGCTGCAGGAGATGATTGA CGAGGTCGATGTAGCCATCAG	179
*IGFBP4*	XM_048053219.1	AGCATCCATGACCGCAAATG CTGTTGCGCATCTTGTTGCC	74
*ACTC1*	XM_048058696.1	CCAGGGTGTTATGGTTGGCA	218
		TAGGGTTCAAGGGGGCTTCT	
*SERPINB5*	XM_013187398.2	ACTCACCCCCGAGACACTAT	120
		TCCAGAAGTGGCTTCAGGTC	
*TTN*	XM_048061293.1	CAAAGCACCTCCACGAATGC CTGTCGTGCCATTTGAGCTT	88
*MYO7B*	XM_013188025.2	GAGGGACGCATTTGTGAAGG GGTGCACGAAGAACTGCTGA	221
*SERPINA1*	XM_013177486.2	GTCCAGCTGAGTATGGGCAA GTCAACAAATTTGCCATGGGT	186
*INS*	XM_013191620.2	GGCTCTCTACCTGGTGTGTG TCCTCATGTTGGAACGGCAG	129
*MYH7B*	XM_048080687.1	AGCCCCGTCCGGATAAAAAG	164
		GGCCAGAAGCTTGTTTTGGG	
*SCD5*	XM_013190576.2	TGTGCTTTGTGATCCCCACC AGGAAGTAGGCGTTCCACAG	70
*FGF10*	XM_013181803.2	TGTCTTCTGTGCCTGTCACC ATGCTGAAGGGGCAGTTCTC	262
*GNA14*	XM_013195956.2	GGAGGGAGTACCAGCTCTCA TGGCACAAAGGAGGGCATAG	80
*MyoG*	XM_048070946.1	CGCCGCCTGAAGAAGGTGAA CCTGCTGGTTGAGGGTGCTGA	154
*MyoD*	XM_013177726.2	CATCCGCTACATCGAGAGCC ACTCCATCATGCCATCGGAG	134
*MYF5*	XM_048078887.1	TGCAAAGCCTGCAAGAGAAAG GTCTCAAACGCCTGGTTCAC	101
*MRF4*	XM_048078889.1	GGTTGTTCCTCGGGGTGTTTTC CTCCTTCTCCCCGTCCAAGTAG	127
*Pax3*	XM_048063046.1	TTCACCTCAGGTAATGGGACTCTTG TGTAGGCAGGCTGTGTAAACTCTTCA	169
*Pax7*	XM_048048726.1	CCTGGGCGACAAAGGTAA GCTCAGCGGTGAAAGTGG	110
*MSTN*	XM_013178647.2	GGCTCTTGATGACGGTAG CTTGTTCCAGACGCAGTT	143

## Data Availability

The data supporting the conclusions of this article are included within the article.
